# Effect of Bariatric Weight Loss on the Adipose Lipolytic Transcriptome in Obese Humans

**DOI:** 10.1155/2015/106237

**Published:** 2015-11-18

**Authors:** Shakun Karki, Melissa G. Farb, Samantha Myers, Caroline Apovian, Donald T. Hess, Noyan Gokce

**Affiliations:** ^1^Evans Department of Medicine and Whitaker Cardiovascular Institute, Boston University School of Medicine, Boston, MA 02118, USA; ^2^Department of General Surgery, Boston University School of Medicine, Boston, MA 02118, USA

## Abstract

*Background.* Dysregulated lipolysis has been implicated in mechanisms of cardiometabolic disease and inflammation in obesity.* Purpose*. We sought to examine the effect of bariatric weight loss on adipose tissue lipolytic gene expression and their relationship to systemic metabolic parameters in obese subjects.* Methods/Results*. We biopsied subcutaneous adipose tissue in 19 obese individuals (BMI 42 ± 5 kg/m^2^, 79% female) at baseline and after a mean period of 8 ± 5 months (range 3–15 months) following bariatric surgery. We performed adipose tissue mRNA expression of proteins involved in triglyceride hydrolysis and correlated their weight loss induced alterations with systemic parameters associated with cardiovascular disease risk. mRNA transcripts of adipose triglyceride lipase (ATGL), hormone-sensitive lipase (HSL), and lipid droplet proteins comparative gene identification 58 (CGI-58) and perilipin increased significantly after weight loss (*p* < 0.05 for all). ATGL expression correlated inversely with plasma triglyceride (TG), hemoglobin A1C (HbA1C), and glucose, and HSL expression correlated negatively with glucose, while CGI-58 was inversely associated with HbA1C.* Conclusion*. We observed increased expression of adipose tissue lipolytic genes following bariatric weight loss which correlated inversely with systemic markers of lipid and glucose metabolism. Functional alterations in lipolysis in human adipose tissue may play a role in shaping cardiometabolic phenotypes in human obesity.

## 1. Introduction

Obesity and its associated widespread metabolic abnormalities such as insulin resistance and dyslipidemia have emerged as major public health problems worldwide [1, 2]. Among the multiple mechanisms responsible for mediating obesity-related cardiovascular disease is the upregulated concentrations of circulating free-fatty acids (FFA) that have been associated with insulin resistance and inflammation [3–5]. Fatty acids play important physiological roles in energy metabolism while also serving as signaling molecules, and their mobilization from triglycerides (TG) is regulated by specific hydrolytic lipases including adipose triglyceride lipase (ATGL) and hormone-sensitive lipase (HSL), as well as lipid droplet proteins comparative gene identification protein 58 (CGI-58) and perilipin.

Experimental studies suggest that functional alterations in the enzymatic activity of lipases under obese conditions may lead to dysregulated FFA metabolism. ATGL protein and mRNA are downregulated in mouse models of obesity [6]. Overexpression of ATGL specific to adipose tissue promotes fatty acid use and attenuates diet-induced obesity [7], while ATGL deficiency leads to changes in insulin signaling and ectopic fat accumulation in nonadipose tissue [8–10]. However, expression of lipases in human obesity is incompletely studied [11–13]. One report demonstrated that both lean and obese subjects express comparable amounts of ATGL protein while HSL is attenuated in obesity [11]. Conversely, another study suggested that although obese subjects express significantly high ATGL mRNA, protein expression is reduced. Moreover, they reported that HSL mRNA was upregulated in visceral but not subcutaneous fat in obesity [12]. Another investigation observed significant reductions in both ATGL and HSL mRNA and protein in obese insulin resistant subjects, and weight loss by hypocaloric diets induced decreases in ATGL and HSL expression [13]. The literature thus demonstrates variable transcriptomic signatures in largely cross-sectional comparisons between different groups of individuals, and limited data are available on longitudinal effects of weight reduction.

Bariatric surgery is currently the most effective and durable method for sustained weight loss and cardiometabolic benefit for the treatment of obesity [14–16]. In the present study, we sought to examine the effect of bariatric surgery on adipose tissue expression of lipolytic enzymes before and after extensive weight loss in obese humans and determine whether tissue changes associate with systemic markers of whole body metabolism.

## 2. Materials and Methods

### 2.1. Study Subjects

Consecutive obese men and women (BMI ≥ 35 kg/m^2^, age ≥ 18 years) with long-standing obesity enrolled in the Boston Medical Center Bariatric Surgery Program were recruited into the study. Subcutaneous adipose tissue samples at baseline were collected intraoperatively from the lower abdominal wall during planned bariatric surgery, as previously described [17–19]. Follow-up fat tissue biopsy was performed percutaneously via periumbilical punch and needle biopsy of subcutaneous fat during a postoperative follow-up visit. The subcutaneous depot that is sampled intraoperatively is the same anatomic layer that is accessed in our follow-up transcutaneous biopsy. Each subject provided two biopsy specimens from the subcutaneous depot, one at baseline and one during the postoperative visit. All biopsies were performed under fasting conditions. Subjects with unstable medical conditions such as active coronary syndromes, congestive heart failure, systemic infection, acute illness, malignancy, or pregnancy were excluded. The study was approved by the Boston University Medical Center Institutional Review Board and written consent was obtained from all participants.

### 2.2. Percutaneous Adipose Tissue Biopsy

For follow-up adipose tissue biopsies, subjects were placed in supine position with sterile draping of the abdominal region. Local skin anesthesia was performed with subcutaneous lidocaine injection and a small superficial 0.5 cm skin incision made lateral to the umbilicus with a tiny scalpel which allows for both aspiration of fat using a large-bore cannula and several punch biopsies and/or manual debridement of tissue below the skin layer, providing specimens of intact adipose tissue. The anatomic layer and qualitative yield of this procedure are the same as the intraoperative baseline collection. The superficial skin incision was then closed with self-absorbing sutures and biopsy sites were inspected in follow-up clinic within 1 week.

### 2.3. Anthropometric and Biochemical Measures

During a presurgical outpatient and subsequent postoperative follow-up visit, clinical characteristics including blood pressure, height, weight, body mass index (BMI), and waist circumference were measured, and cardiovascular risk factors were recorded. Fasting blood was taken via an antecubital vein for biochemical measures including lipids, glucose, insulin, glycosylated hemoglobin (HbA1c), high-sensitivity CRP (hs-CRP), and homeostasis model assessment (HOMA) as the index of insulin sensitivity. All biochemical analyses were performed by the Boston Medical Center clinical chemistry laboratory.

### 2.4. Adipose Tissue Gene Expression

Immediately following adipose tissue collection, tissue samples were stored in RNAlater (Sigma Aldrich) solution at −80°C. Total RNAs were isolated from homogenized whole adipose tissues using the QIAzol reagent and RNeasy Mini kits (Qiagen, Germantown, MD). RNA (0.5–1.5 *μ*g) was retrotranscribed with High Capacity cDNA Synthesis Kits (Life Technologies). Quantitative real time PCR reactions were performed using TaqMan gene expression assays in a ViiA7 PCR system (Life Technologies). Results were analyzed with the ΔΔCt method using GAPDH as a reference.

### 2.5. Statistics

Clinical characteristics of subjects were analyzed using SPSS 20.0 and presented as mean ± SD or percentage. All other analyses were performed using GraphPad Prism 6.0 software. Differences in clinical characteristics and gene expression between baseline and follow-up visits were examined using Student's paired *t*-tests. Spearman correlation analysis was performed to examine associations between lipolytic gene expression and clinical parameters which were normally distributed. A value of *p* < 0.05 was accepted as statistically significant. Graphic data are presented as mean ± SEM unless otherwise indicated.

## 3. Results

### 3.1. Clinical Characteristics

A total of 19 obese (BMI 42 ± 5 kg/m^2^, 79% female) subjects were enrolled and followed longitudinally for a mean period of 8 ± 5 months (range 3–15 months) after bariatric surgery.  Table 1 displays the clinical characteristics of subjects at baseline and after weight loss. As expected, bariatric intervention produced a significant 25% weight decline for the entire group. This was associated with significant decreases in BMI, waist circumference, HbA1c, triglycerides, HOMA-IR, insulin, glucose levels, and prevalent hypertension. Additionally, there was a marked decrease in hs-CRP as a marker of systemic inflammation with >5-fold decline following bariatric surgery.

### 3.2. Adipose Tissue Gene Expression

**Figure 1:**
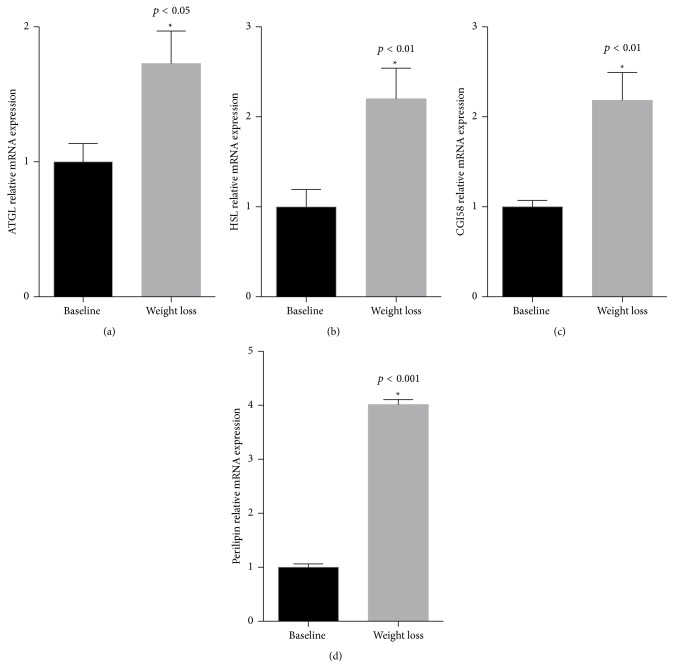
Lipolytic gene expression before and after bariatric surgery. Lipolytic gene mRNA for ATGL ((a), *p* < 0.05), HSL ((b), *p* < 0.01), CGI-58 ((c), *p* < 0.01), and perilipin ((d), *p* < 0.001) significantly increased in subcutaneous adipose tissue following bariatric surgery. Data are presented as relative expression, mean ± SEM, *n* = 19.

As shown in Figure 1, relative mRNA expression of ATGL (a), HSL (b), CGI-58 (c), and perilipin (d) significantly increased after weight loss surgery compared to baseline, with greatest relative increase observed for perilipin.

### 3.3. Correlations between Lipolytic Gene Expression and Clinical Parameters

**Figure 2:**
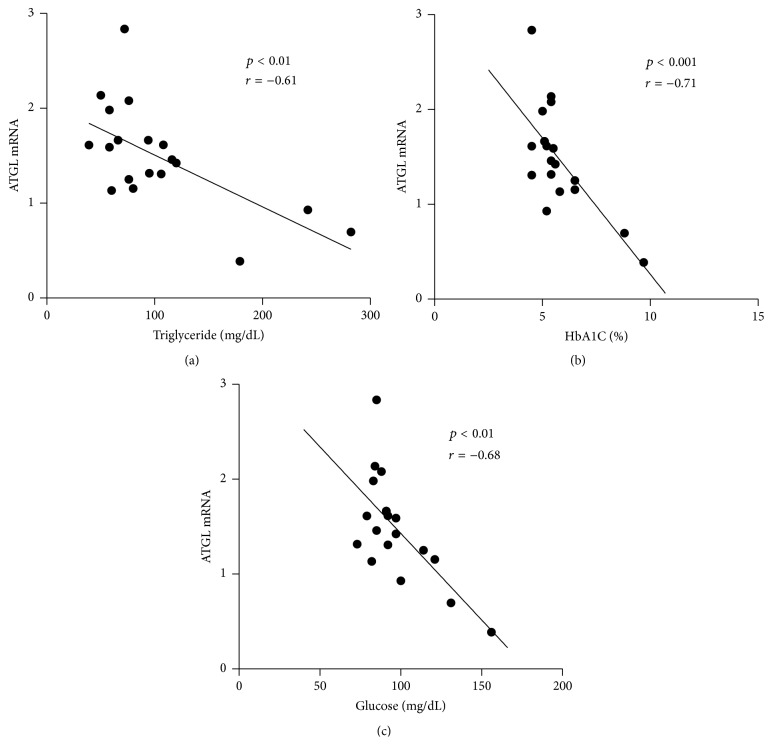
Correlations between ATGL mRNA and clinical parameters. (a) ATGL mRNA expression inversely correlated with plasma triglycerides after weight loss (*p* < 0.01, *r* = −0.61). (b) ATGL mRNA inversely correlated with plasma HbA1C (*p* < 0.001, *r* = −0.71). (c) ATGL mRNA was negatively associated with fasting blood glucose (*p* < 0.01, *r* = −0.68).

**Figure 3:**
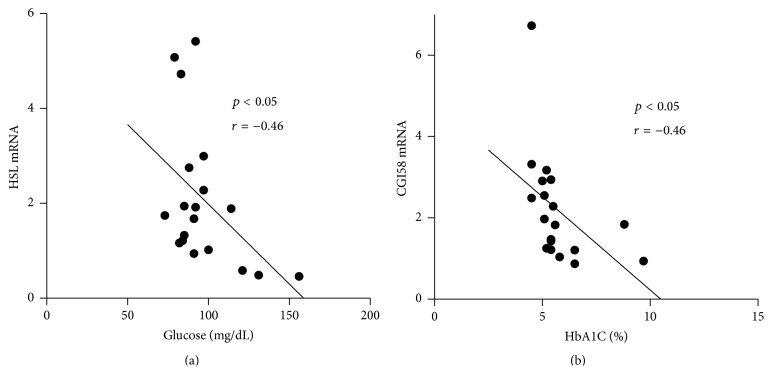
Associations between HSL, CGI-58 mRNA, and clinical parameters. (a) HSL mRNA expression negatively correlated with fasting blood glucose level after weight loss (*p* < 0.05, *r* = −0.46). (b) CGI-58 mRNA was negatively associated with HbA1C (*p* < 0.05, *r* = −0.46).

Plasma concentration of TG (Figure 2(a)), HbA1C (Figure 2(b)), and glucose (Figure 2(c)) correlated negatively with ATGL mRNA expression after weight loss. In addition, plasma glucose was inversely associated with HSL mRNA (Figure 3(a)), with a similar finding trending for HbA1C (*p* = 0.08, data not displayed). Gene expression of CGI-58 gene was inversely correlated with plasma HbA1C after weight loss (Figure 3(b)). We found no correlations between adipose gene expression and clinical parameters at baseline (data not displayed).

## 4. Discussion

In the present study, we longitudinally examined the effect of surgical weight loss on subcutaneous adipose tissue transcripts of lipases and lipid droplet proteins involved in triglyceride hydrolysis and metabolism in obese humans. mRNA transcripts of ATGL, HSL, CGI-58, and perilipin significantly increased following weight loss, and their expression correlated inversely with systemic metabolic parameters including plasma triglycerides, glucose, and HbA1C. These findings suggest that weight decline is associated with lipolytic alterations that are detectable in human adipose tissue and are linked to processes that may regulate systemic metabolism.

Adipose tissue serves as an energy reservoir that modulates triglyceride clearance and FFA release in response to whole body metabolic requirements. Under conditions of obesity and positive energy balance, fat accumulates in both adipose tissue and ectopic organs and is associated with the development of dyslipidemia, insulin resistance, and inflammation in both animal models and humans [17, 20–23] which increase cardiovascular disease risk. These processes may involve dysregulation of several enzymes including ATGL and HSL and cofactors. Although the published literature is mixed on the relative expression of ATGL in human adipose tissue in obesity [11–13, 24], we demonstrated consistent increases in expression following weight loss which suggests downregulation under obese conditions. Weight decline would presumably stimulate lipolysis and thus increase enzymatic activity in human fat stores. Moreover, we observed that this process is associated with improved insulin resistance possibly owing to decreased FFA flux, as ATGL has been linked with increased insulin sensitivity [25] and increased plasma FFA mobilization [26].

Data on HSL activity and expression in obesity have also been mixed. There are reports of decreased HSL lipolytic activity with obesity [11], and possible gender differences with decreased or unaltered HSL protein with weight loss [27, 28]. We now demonstrate consistent increases in HSL mRNA, as with ATGL, following weight loss which correlated inversely with plasma glucose. We also observed upregulation of perilipin and CGI-58 which represent key proteins associated with intracellular lipid droplets. Mutations in CGI-58 in animal models lead to deficient catabolism of cellular triacylglycerol and promote lipid accumulation in nonadipose tissue [29]. Moreover, patients with mutations in CGI-58 exhibit defective lipolysis and ectopic lipid accumulation in multiple tissues [30]. It is believed that efficient ATGL activity requires CGI-58 which associates with lipid droplets and interacts with perilipin that modulates droplet turnover [31]. Our observation of a coordinate upregulation of all four measured transcripts associated with lipolysis and lipid droplets following weight loss suggests that their activities may be interlinked for effective triglyceride hydrolysis and consequently whole body metabolism, as supported by significant correlations with systemic measures of triglyceride and glucose handling.

In the present study, we also observed a remarkable decline in plasma hs-CRP which has been described previously in association with weight loss [32, 33]. It is well established that obesity is associated with a chronic, subclinical degree of inflammation that is derived in part from macrophage-driven adipose tissue inflammation in response to several pathological tissue changes including adipocyte hypertrophy [21, 34]. Local overproduction of FFA may also represent a mediator for immune activation [3, 4, 35, 36] and our observation of reduced inflammation with weight loss may be related, in part, to decreased FFA following weight loss as previously established [37, 38], although this was not specifically measured in this study.

There are several limitations to our study. The sample size is relatively small and experimental design was limited to a surgical population undergoing bariatric surgery and thus findings may not be applicable to the general population or lesser degrees of obesity. However, our demonstration of significant clinical correlations even with this small sample makes our results more compelling. Moreover, our findings are limited to the subcutaneous depot. We acknowledge that the visceral depot may be more metabolically active compared to subcutaneous one; however sequential visceral biopsies are not possible since a repeat invasive abdominal operation would be required which is not justifiable for only research purposes. However, we believe that much can be learned from examining longitudinal changes in the subcutaneous fat of obese individuals. Additionally, we acknowledge that methods for collecting fat samples at baseline and follow-up biopsy were different and may have affected gene expression; however we believe that the techniques which are all performed under fasting conditions are comparable. We did not measure protein levels or examine functional activity of these enzymes; thus observations are limited to mRNA expression patterns; however protein tracked mRNA transcripts in a prior study [13]. Lastly, our study design does not enable us to distinguish between the effect of negative energy balance and the effect of weight loss on changes in gene expression and clinical parameters. These limitations are counterbalanced by our ability to study the same subjects longitudinally by examining the effect of major weight loss and studying changes in adipose tissue that may provide clues to mechanisms of systemic disease.

In conclusion, we observed increased expression of adipose tissue lipolytic genes which correlated inversely with systemic markers of lipid and glucose metabolism following bariatric weight loss. Functional alterations in lipolytic activity in human adipose tissue may play important roles in shaping systemic phenotypes associated with cardiovascular risk factors in human obesity.

## Figures and Tables

**Table 1:** Clinical characteristics.

Parameter	Baseline	Weight loss	*p* value
BMI (kg/m^2^)	42 ± 5	32.2 ± 6	<0.001
Waist circumference (cm)	123 ± 12	91 ± 37	<0.01
Weight (kg)	114 ± 14	85 ± 14	<0.001
Insulin (mIU/mL)	26.6 ± 34	11.4 ± 14	<0.001
Glucose (mg/dL)	139 ± 99	98 ± 21	<0.05
HbA1C (%)	6.6 ± 2.3	5.8 ± 1.4	<0.05
HOMA-IR	4.6 ± 8.4	3.1 ± 5	<0.05
hsCRP (mg/dL)	10.6 ± 9.7	2.0 ± 2	<0.05
Triglycerides (mg/dL)	119 ± 81	91 ± 37	<0.05
HDL-C (mg/dL)	51 ± 14	56 ± 16	0.31
LDL-C (mg/dL)	117 ± 36	102 ± 34	0.12
Systolic BP (mmHg)	126 ± 14	127 ± 15	0.66
Diastolic BP (mmHg)	73 ± 14	77 ± 11	0.71
Diabetes (%)	21	11	0.07
Hypertension (%)	40	13	<0.05
Hypercholesterolemia (%)	16	5	0.08

Data are mean ± SD. *n* = 19.
